# Study of Sexual-Linked Genes (*OGI* and *MeGI*) on the Performance of Androecious Persimmons (*Diospyros kaki* Thunb.)

**DOI:** 10.3390/plants10020390

**Published:** 2021-02-18

**Authors:** Liyuan Wang, Weijuan Han, Songfeng Diao, Yujing Suo, Huawei Li, Yini Mai, Yiru Wang, Peng Sun, Jianmin Fu

**Affiliations:** 1 Key Laboratory of Non-Timber Forest Germplasm Enhancement & Utilization of State Administration of Forestry and Grassland, Non-Timber Forestry Research and Development Center, Chinese Academy of Forestry, Zhengzhou 450003, China; wangliyuan1120@163.com (L.W.); hanweijuan2013@163.com (W.H.); dsf@caf.ac.cn (S.D.); suoyj@caf.ac.cn (Y.S.); lihuaweicaf@163.com (H.L.); maiyini94@sina.com (Y.M.); wangyiru199702@163.com (Y.W.); 2 Research Institute of Forestry, Chinese Academy of Forestry, Beijing 100091, China

**Keywords:** *Diospyros kaki*, androecious persimmons, *OGI*, *MeGI*, sex-determining mechanism

## Abstract

It is reported that the production of floral sexual phenotype in hexaploid monoecious persimmon (*Diospyros kaki*) is closely related to a pseudogene called *OGI*, and a short interspersed nuclear element (SINE)-like insertion (named *Kali*) in the *OGI* promoter leads to the gene silence. As a result, DNA methylation level of *MeGI* promoter determines the development of male or female flowers. However, the molecular mechanism in androecious *D. kaki*, which only bear male flowers, remains elusive. Here, real-time quantitative polymerase chain reaction (RT-qPCR), molecular cloning, and bisulfite PCR sequencing technique were carried out using 87 materials, including 56 androecious resources, 15 monoecious, and 16 gynoecious cultivars, to investigate the performance of *OGI* and *MeGI* on the specific androecious type of *D. kaki* in China. In conclusion, the *Kali* insertion was exactly located in the *OGI* promoter region, and the *OGI* gene and the *Kali* sequence were existing and conserved in androecious *D. kaki*. Meanwhile, we also demonstrated that the *MeGI* gene was widespread in our investigated samples. Ultimately, our result convincingly provided evidence that the low expression of *OGI* is probably ascribed to the presence of *Kali* displaying strong methylation in the *OGI* promoter, and low expression of *MeGI,* as well as high DNA methylation level, in the promoter was closely connected with the production of male flowers; this result was consistent with the monoecious persimmon model. Our findings provide predominant genetic aspects for investigation into androecious *D. kaki*, and future perfecting the sex-determining mechanisms in persimmon.

## 1. Introduction

*Diospyros kaki* Thunb. (*D. kaki*) is an economically important fruit tree species in China [1,2,3]. There were three types of *D. kaki* at the individual level sex expression: (i) bear only female flowers (gynoecious-type), (ii) both pistillate and staminate flowers on the same plant (monoecious-type), and (iii) hermaphroditic, pistillate, and staminate flowers on the same plant (polygamomonoecious-type) [4]. In addition, a type bearing only male flowers (androecious-type) was found in the Dabie Mountain area in China [5,6]. *D. kaki* shows flexible sexuality characteristics, and the reason has not yet been fully elucidated.

In the dioecious diploid persimmon, *Diospyros lotus* (*D. lotus*), which has the most closely genetic relationship with *D. kaki*, the floral sexual phenotype was determined by two genes called *OGI* (Japanese for “male tree”) and *MeGI* (Japanese for “female tree”). Briefly, the autosomal *MeGI* gene encodes a homeodomain transcription factor and promotes the development of female flowers, whereas the Y-encoded pseudogene *OGI* encodes small-RNAs targeting and repressing the *MeGI* gene and is responsible for the formation of male flowers [7]. In monoecious hexaploid *D. kaki*, the *OGI* gene exists [8], but its expression is silenced by a DNA sequence called *Kali*. The *Kali* was located in the *OGI* promoter and exhibited high homology to a short interspersed nuclear element (SINE)-like retrotransposon element.

Both *OGI* gene and *MeGI* gene could be found in monoecious persimmons, but there was only *MeGI* existing in gynoecious trees [8]. Based on the research above, Akagi et al. [9] investigated the epigenetic regulation of the sex determinate gene *OGI* and *MeGI* in *D. kaki* and found that the production of male and female flowers was determined by the methylation levels of *MeGI*, especially in the 250 bp immediately upstream of the *MeGI* start codon [9]. The finding provides great significance for the sex determination in monoecious *D. kaki*. According to the above situation, using androecious genotype patterns and some F1 progeny, Zhang et al. [10] claimed that the *OGI* gene marker could be used to distinguish *D. kaki* sexuality at an early stage. However, the molecular mechanism of sex determination in androecious types of *D. kaki* remains elusive due to the limited number of androecious individual available. Our group made the wild germplasm resources survey of persimmon from 2012 to 2017 nationwide, and found the distribution of male persimmons in Yunjia mountain and Mulan mountain from Hubei province, Guilin from Guangxi Zhuang Autonomous Region, Suzhou from Jiangsu province, etc. [11]. According to the current results, we highlighted the mechanisms of the sexuality of androecious *D. kaki*, and the following questions need further investigation: (1) whether androecious *D. kaki* contains *kali* in the promoter of *OGI*? (2) Whether the *kali* and *OGI* sequences were existing and conserved in male flowers of androecious *D. kaki*? (3) Is there any relation between the *OGI* gene expression and the methylation level of *Kali*? (4) Is the development of male floral buds of androecious *D. kaki* controlled by the methylation level of *MeGI* promoter? (5) If the answers to all the questions are same as that in monoecious *D. kaki*, what factors are responsible for the difference of sexuality between monoecious and androecious *D. kaki*?

In the present study, we make full use of the specific materials of androecious *D. kaki* to study the expression of *OGI* and *MeGI*, and compare the results to the findings reported by Akagi et al. [9] and Zhang et al. [10]. Hence, this finding will contribute to improving the *D. kaki* gender theory model, meanwhile providing the foundation for appropriate management during the flowering period, and having the potential to cultivate excellent male germplasm which is valuable in cross-breeding work.

## 2. Results

### 2.1. Kali Was Confirmed to Be Located in the Promoter Region of OGI

**Figure 1:**
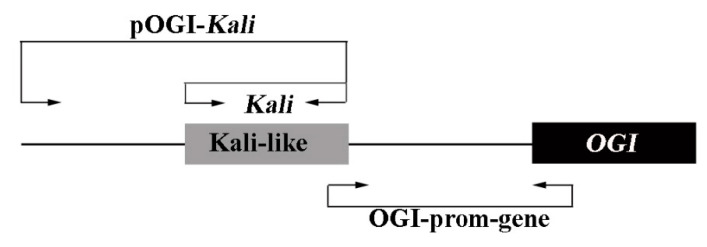
Primer sets of the promoter region and the *OGI* gene.

Schematic structure of the promoter region and the *OGI* gene showed that the *Kali* was designed to specifically amplify the *Kali* region; the pOGI-Kali was for part of the region of the *OGI* promoter, including the *Kali*, and the OGI-prom-gene was for the latter part of *Kali* following the former part of the *OGI* gene region (Figure 1).

To validate that the amplicon of *Kali* was just located in the promoter region of *OGI*, a pair of primers targeting pOGI-Kali was used for PCR, and the fragments amplified from seven androecious samples were cloned into *Escherichia coli* and sequenced. It is satisfied that the *Kali* was exactly located in the end of our obtained sequences of pOGI-Kali (Figure S1). Indeed, we also designed a pair of primers named OGI-prom-gene, which consistently amplified the sequences from the latter part of *Kali* to the front part of *OGI*, and the result from the seven androecious samples showed that the DNA fragments (File S1) could be connected with the pOGI-Kali and *OGI* sequences.

### 2.2. Kali and OGI Were Existing in Androecious D. kaki

PCR analysis revealed that the *Kali* was present in all androecious *D. kaki* samples tested, as well as the monoecious and gynoecious cultivars. In addition, the pOGI-Kali sequence was not found in 16 gynoecious cultivars, but was present among the 50 androecious and 10 monoecious individuals. Similarly, the specific PCR fragments confirmed the presence of *OGI* in a wide variety of *D. kaki* bearing male flowers (including androecious and monoecious individuals), and the absence in the gynoecious cultivars. All samples tested here demonstrate that the *Kali* exists in all types of *D. kaki*, whereas *OGI* is just found in androecious and monoecious *D. kaki* (Table 1).

### 2.3. Kali and OGI DNA Sequences Were Conserved

To characterize variation about the amplicons in *D. kaki* resources of androecious and the fragments obtained from monoecious by Akagi et al. [9], seven *Kali* fragments obtained from seven androecious samples were cloned into *E. coli* and sequenced. DNA sequence alignment analysis showed that over 94% identity was observed (Figure 4), suggesting the *Kali* sequence is conserved in *D. kaki* samples bearing male flowers (including androecious and monoecious persimmons).

In the sequencing of our seven *OGI* fragments, 54–57 termination codons consisting of 17.65–18.79% bases were found, and TAG was the most common type, followed by TAA (Table 2). It was also observed that the compositions of bases in *OGI* sequences derived from our *D. kaki* samples displayed a trend of A > T > G > C. To further investigate the universality, the *OGI* sequences were compared with the fragments obtained from Zhang et al. [10]. DNA sequence alignment analysis of *OGI* among androecious and monoecious *D. kaki* individuals revealed that the *OGI* sequence identity was over 95% (Figure 5), confirming the sequence is conserved and indispensable in androecious persimmons.

### 2.4. Expression of OGI and Methylation Level of Kali

**Figure 2:**
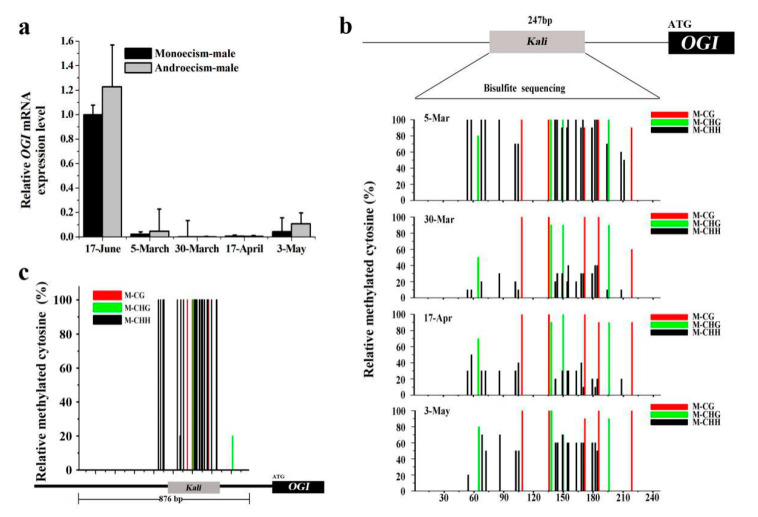
Expression of *OGI* and methylation level of *Kali*. (**a**) RT-qPCR analysis of the relative *OGI* mRNA expression level during different developmental stages (Table 1, sample list); (**b**) DNA methylation of the *Kali* sequence from developing bud/flower at different developmental stages in androecious *D. kaki* of Yunjiashan-3; (**c**) DNA methylation across the *OGI* promoter from male flowers in androecious *D. kaki* of Jiangxi Yeshi-1 at the stage of 3 May. M-CG, M-CHG, and M-CHH represent different sequence context of cytosine methylation. Values are means ± SE (N = 14 for monoecious biological replicates; N = 49 for androecious biological replicates).

In male flowers of androecious *D. kaki* trees, the expression trend of *OGI* was basically in accordance with that of monoecious *D. kaki*, at least at the bud/flower different developmental stages in our study (Figure 2a). The *OGI* levels in androecism-male on 17 June and 3 May were slightly higher than that in monoecism-male. Meanwhile, we also observed no numerous, or rather, low-expressed *OGI* mRNA at the stages of 5 March to 17 April.

Next, an androecious *D. kaki* of Yunjiashan-3 was sampled for the investigation of DNA methylation of the *Kali* sequence, and Figure 2b proves that the developing bud/flower at different developmental stages all presented different levels of methylation, which corresponded with the low expression of *OGI*. Notably, dramatically higher methylation levels were visible in *Kali* sequence compared with the other regions across the *OGI* promoter from another androecious *D. kaki* of Jiangxi Yeshi-1 at the stage of May 3 (Figure 2c). All in all, the *Kali* insertion of the *OGI* promoter upstream exhibited strong cytosine methylation along its entire length in developing buds and flowers, implying the repressive role on the *OGI* expression.

### 2.5. MeGI mRNA Expression Level and Promoter Methylation

**Figure 3:**
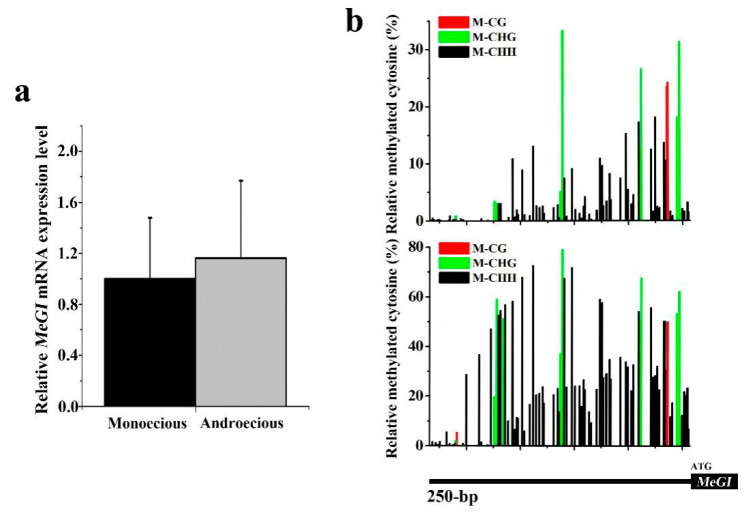
Expression of *MeGI* and methylation level of its promoter. (**a**) RT-qPCR analysis of the relative *MeGI* mRNA expression level on 17 April (Table 1, sample list); (**b**) cytosine methylation levels in the 250 bp immediately upstream of the *MeGI* promoter in male flowers on 17 April from androecious *D. kaki* of Mulan-7 (upper panel) and Mulan-24 (lower panel). M-CG, M-CHG, and M-CHH represent different sequence context of cytosine methylation. Values are means ± SE (N = 14 for monoecious biological replicates; N = 49 for androecious biological replicates).

**Figure 4:**
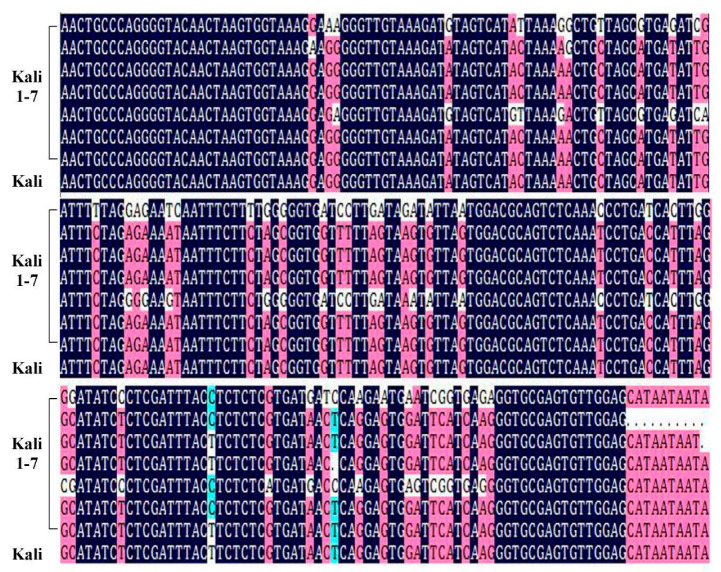
Alignment of the *Kali* sequences in *Diospyros kaki* genotypes bearing male flowers. *Kali* 1–7 represent the *Kali* sequences of Yunjiashan-8, Mulan-38, Luotian Yeshi-1, Jiangsu Yeshi 2, Jiangxi Yeshi-1, Hunan Yeshi-1, and Yunjiashan-3, respectively, which were all obtained from our androecious *D. kaki* genotypes, and the ‘*Kali*’ was from monoecious genotype, i.e., Taishu, from Akagi et al. [8].

**Figure 5:**
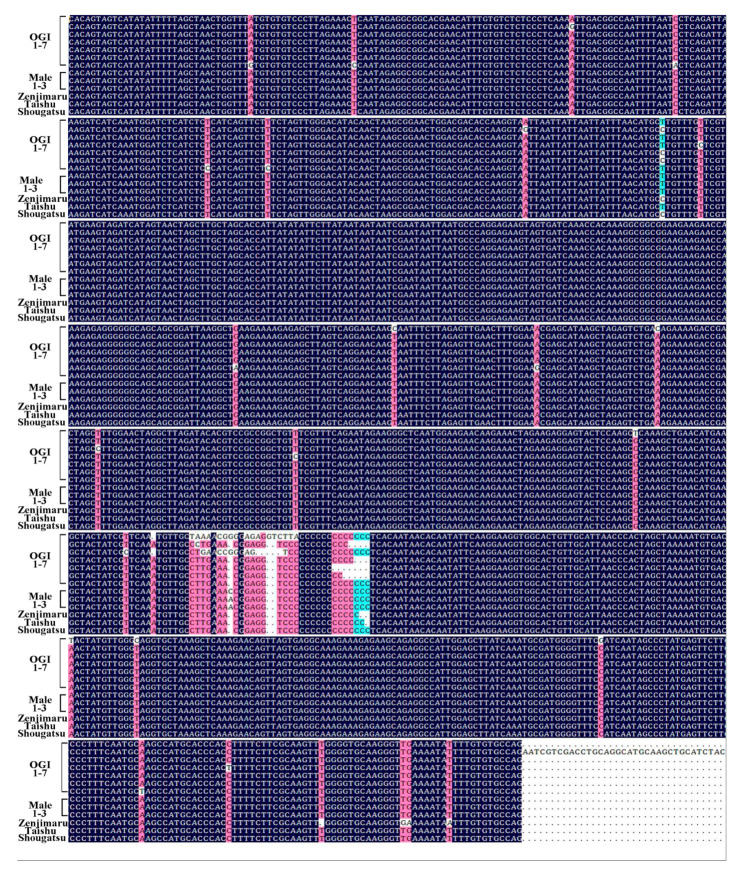
Alignment of the *OGI* sequences in *D. kaki* bearing male flowers. *OGI* 1–7 represent the *OGI* sequence of Yunjiashan-8, Mulan-38, Luotian Yeshi-1, Jiangsu Yeshi 2, Jiangxi Yeshi-1, Hunan Yeshi-1, and Yunjiashan-3, respectively, which were all obtained from our androecious *D. kaki*. Male 1–3 represent androecious *D. kaki* isolate plant 1–3, and Zenjimaru, Taishu, and Shougatsu were monoecious *D. kaki*, which were all obtained from Zhang et al. [10].

To better understand the correlation between the gene expression and sex types in androecious *D. kaki*, the *MeGI* gene at the stage of 17 April, when female primordia arrested in male floral buds, and male primordia arrested in female floral buds, was further analyzed. As shown in Table 1, PCR product of *MeGI* gene was widespread in androecious *D. kaki*, which was the same as monoecious ones, and no significant difference of *MeGI* expression level was determined between male flowers from these two *D. kaki* types (Figure 3a). Meanwhile, 0–40% or even 80% of methylation levels in the 250 bp immediately upstream of the *MeGI* start codon were visible in androecious male flowers, and we detected all three methylation contexts (CG, CHG, and CHH), among which, the CHH sequence was identified as the most widespread context (Figure 3b).

## 3. Discussion

Epigenetics, an integral part of the genetics, is defined as changing the expression of genes, but the changing is reversible and heritable while maintain the DNA sequence of gene [12,13]. There are many categories of epigenetics being documented, and DNA methylation and non-coding RNA regulation are the most widely modified of genomes [14]. DNA methylation generally occurs in highly-repetitive DNA sequences such as transposons, promoter, and gene-encoding areas. It is essential to plant development and evolution, by interacting with transcription factors or affecting chromatin structure and DNA conformation to regulate the genetic information at the epigenetic level [15]. DNA methylation exists in CG, CHG, and CHH sequence contexts (where H is A, C, or T). The CG methylation is common in expressed gene bodies, while all the sequence contexts (including CG, CHG, and CHH methylation) located in gene promoter could lead to gene silence [16,17]. Meanwhile, the establishment of DNA methylation is mainly through a pathway of the RNA-directed DNA methylation (RdDM), and siRNA plays an irreplaceable role in the process [18]. Non-coding RNAs, such as microRNA, long non-coding RNAs, and circular RNAs, are a vast and heterogeneous family of RNAs; they are involved in cell differentiation and act in modulating translation and transcription of target genes, thus leading to epigenetic changes [19,20]. For example, miRNA160, miRNA396, miRNA535, and miRNA5021 regulate cell division and differentiation in yam during its expansion stage [21]. doublesex1 alpha promoter-associated long RNA (*DAPALR*) regulates the male-determining gene (*Dsx1*) expression in the *Daphnia magna* [22]. Although the actual mechanism underlying how DNA methylation and non-coding RNA trigger the initiation of epigenetic modification in organic evolution process is unknown, there is no doubt that they could enhance gene expression or inhibit gene silence at the transcript level, so as to play a central role in plant growth and development.

Recent studies have already recorded some sex determination sites on sex chromosomes in a small part of dioecious plants. The sex determination site of spinach (*Spinacia oleracea*) was located on the N3 linkage group [23], the poplar was on chromosome 19 [24], the asparagus was on chromosome 5 [25], and the papaya (*Carica papaya*) was on the N1 linkage group [26]. Additionally, in respect of gene, it was demonstrated that *CmACS-7* and *CmWIP1* were the sex-determining genes in melons [27], and *CS-ACS2* and *CS-ACS1G* were for cucumber [28]. The roles of *NA1* (associated with BR synthesis) and *TS1* (associated with jasmonic acid synthesis) were identified as the sex-determining genes in the monoecious maize (*Zea mays*) [29,30]. Although the sex chromosome on plants has been studied for more than a century, the identified sex determination genes in dioecious trees were extremely limited [31], presumably because of the shorter evolution time and the restructure of mutations between chromosomes. At present, one gene, namely *OGI*, was validated as the sex-determining gene in *D. lotus*, which regulates sexual differentiation by targeting the autosomal *MeGI* gene through encoding small-RNAs [7]. In monoecious *D. kaki*, the *OGI* is silenced, and DNA methylation level of the *MeGI* promoter determines the development of male or female flowers [8].

In our study, seven *OGI* fragments derived from androecious *D. kaki* were cloned into *E. coli* and sequenced. Results of termination codons and compositions of bases in these *OGI* sequences were in accordance with a previous report that the coding sequence of *OGI* presented multiple disruptive stop codons [7]. BLAST analysis revealed that our androecious *OGI* sequences had high sequence identity of over 95% with that of monoecious persimmons, as reported before [10]. Additionally, results of RT-qPCR indicated that the *OGI* gene could have a basal, but not too much, expression level in androecious *D. kaki* bud/flower based on the same primer put forward by Akagi et al. [9]. Overall, we confirmed that this *OGI* DNA sequence is conserved and the expression is low in androecious persimmons.

Indeed, an insertion element named *Kali* in the *OGI* 5′ upstream promoter region exhibiting strong cytosine methylation potentially contributed to the silence of *OGI* expression in monoecious *D. kaki* [9]. To further confirm the existence of *Kali*, we took advantage of the *Kali* and pOGI-Kali primers described by Akagi et al. [9], and convincingly showed that the amplicon was present and conserved in all tested androecious *D. kaki*. Meanwhile, the sequences amplified by a new designed pair of primers called OGI-prom-gene could be connected with the pOGI-Kali and *OGI* region, highlighting that the conserved *Kali* is exactly located in the *OGI* promoter region in all androecious persimmons. Regarding the cytosine methylation, considerably higher levels were observed in the *Kali* region compared with the other parts of the *OGI* promoter in androecious *D. kaki*. In summary, in the specific materials of androecious persimmons, the *OGI* is low-expressed, potentially due to the presence of highly-methylated *Kali* in the promoter. This result was largely concordant with the findings in hexaploid monoecious persimmon documented by Akagi et al. [9]. Thus, other regulatory factors and mechanisms may be responsible for the difference of sexuality between monoecious and androecious *D. kaki*.

More importantly, male flower buds of monoecious *D. kaki* exhibited strong signal of *MeGI* gene methylation levels, which was consistent with the low expression of *MeGI* [9]. In our study, similar *MeGI* levels to that of monoecious *D. kaki,* as well as 0–40% or even 80% methylation of the *MeGI* promoter region in male flowers of androecious *D. kaki,* were recorded. As a result, we deduced that strong methylation levels of the *MeGI* promoter may play a remarkable role in the performance of male flowers. Akagi et al. [9] revealed that RNA-based sex-determining mechanism occurred in monoecious *D. kaki*, for the methylation signal present on the *MeGI* promoter can activate 21 nt long smMeGI production, which was abundant in male buds/flowers, while 24 nt small RNA was high, and accumulated on the *Kali* insertion in the *OGI* promoter. It is thus possible that small RNA encoded by the Y chromosome inhibited the expression of *MeGI* through activating RdDM pathway on the *MeGI* promoter in monoecious *D. kaki*, leading to the male organs. Thus, detailed studies on the bioinformatics characteristics of the *Kali*, as well as the research on the small RNA during the flower bud differentiation process, is sorely needed.

## 4. Materials and Methods

### 4.1. Plant Materials

Eighty-seven *D. kaki* germplasm resources, including 56 androecious individuals, 15 monoecious cultivars, and 16 gynoecious cultivars were sampled in this study (Table 1).

### 4.2. PCR Detection

Fresh leaves from 50 androecious resources, 10 monoecious, and 16 gynoecious cultivars (Table 1) harvested on 3 May (during the anthesis stage) were obtained to determine the existence of *OGI*, *Kali*, pOGI-Kali (amplifies sequence of *OGI* promoter including the *Kali*), and *MeGI* using PCR reaction.

Total genomic DNAs were extracted from fresh leaves using the cetyltrimethylammonium bromide (CTAB) method [32]. PCR was performed using *Taq* PCR Master Mix (Sangon Biotechnology Co., Shanghai, China). The PCR reaction was conducted with the primers provided in Table 3, and the reaction was performed in a programmable T100TM Thermal Cycler (Bio-Rad Laboratories, Hercules, CA, USA) with the following conditions: 3 min for denaturation at 94 °C, followed by 30 cycles at 94 °C for 30 s, annealing at 55 °C for 30 s, 72 °C for 60 s, and final extension at 72 °C for 5 min.

### 4.3. Cloning of the Targeted Fragments

To detect whether the *OGI* and *Kali* sequences were conserved, fresh leaves from seven androecious samples (Table 1) harvested on 3 May were tested by cloning analysis.

Total DNA was extracted from fresh leaves by the CTAB method [32]. Primers specific to *OGI*, *Kali*, pOGI-Kali, and OGI-prom-gene (amplifies the region from the latter part of *Kali* to the former part of *OGI* gene) were displayed in Table 3. PCR amplification was conducted according to the manufacturer’s instructions using P*fu* DNA Polymerase (Sangon Biotechnology Co., Shanghai, China). The products were then detected by 1% agarose gel electrophoresis. After recovering from the gel, the specific PCR fragments were ligated into pUC18-T vector and transformed into *Escherichia coli*. After overnight culture, the single colonies were picked for detection via PCR. The positive clones were sent to Sangon Biotech for sequencing analysis. Alignment sequence was constructed using DNAMAN software (version 7.0; Lynnon Biosoft, San Ramon, CA, USA).

### 4.4. Real-Time Quantitative Polymerase Chain Reaction

To compare expression patterns in 14 androecious and 49 monoecious ones, male buds/flowers (Table 1) were harvested on June 17 of the previous year, and 5 March, 17 April, and 3 May for real-time quantitative polymerase chain reaction (RT-qPCR) analysis. These time periods correspond to the inflorescence primordia initiation (June), petal, carpel, and anther primordia initiation (March), microsporocyte/megasporocyte initiation (April), and mature pollen/embryo sac formation (May). Dynamic changes of *OGI* gene expression during primordia formation and flower development and *MeGI* gene on 17 April in male flowers from androecious and monoecious persimmons were detected.

The total RNA was isolated from male buds/flowers collected across different developmental stages using EZ-10 DNAaway RNA Miniprep Kits (Sangon Biotechnology Co., Shanghai, China) according to the manufacturer’s instructions. The first strand of cDNA was synthesized by a TRUEscript 1st Strand cDNA Synthesis Kit (Kemix, Beijing, China) and RT-qPCR experiments described here were performed with the 2×Sybr qPCR Mix (Kemix) using the CFX96™ Real-Time System (Bio-Rad Laboratories, Hercules, CA, USA), according to the suppliers’ manuals. RT-qPCR analyses of *MeGI* and *OGI* expression (primers in Table 3) were carried out as follows: 3 min at 95 °C for denaturation, followed by 45 cycles of 95 °C for 10 s, 57–60 °C for 5 s, and 72 °C for 15 s. Three technical replicates were performed for each sample, and *GAPDH* was used as the internal control.

### 4.5. Bisulfite PCR Sequencing for OGI Promoter and MeGI Promoter

Male bud/flower from androecious *D. kaki* cv. Yunjiashan-3 at developmental stages of 5 March, 30 March, 17 April, and 3 May, and male flowers from androecious *D. kaki* cv. Jiangxi Yeshi-1 on 3 May were sampled for bisulfite PCR sequencing for *OGI* promoter.

Total DNA was extracted by the CTAB method [32] and purified by phenol/chloroform extraction. Two ug DNA was subjected to bisulfite treatment to deaminate non-methylated cytosine (C) residues into uracil (U) residues. The corresponding non-methylated genome area was identified as the control. The primer set *Kali* (Table 3) was used for bisulfite PCR sequencing of *Kali* sequence, and the eluted DNA was enriched by PCR with the following conditions: 4 min at 95 °C; 35 cycles of 30 s at 94 °C, 30 s at 57 °C, and 1 min at 72 °C; and a final extension step of 8 min at 72 °C. The OGI-prom-F1, OGI-prom-F2, and OGI-prom-F3 were used for bisulfite PCR sequencing of *OGI* promoter (Table 3), and the PCR conditions were 4 min at 98 °C; 20 cycles of 45 s at 94 °C, 45 s at 66 °C (0.5 °C decreased per cycle), and 1 min at 72 °C; 20 cycles of 45 s at 94 °C, 45 s at 56 °C, and 1 min at 72 °C; and a final extension step of 8 min at 72 °C. Afterwards, the specific PCR fragments were ligated into pUC18-T vector and transformed into *Escherichia coli*. After overnight culture and single colonies were picked for detection via PCR, the positive clones were sent to Sangon Biotech for sequencing analysis.

Male flowers of androecious *D. kaki* individuals, including Mulan-7 and Mulan-24 on 17 April, were sampled for detection of methylated cytosine residue for *MeGI* promoter according to the method of Akagi et al. [9]. The bisulfite-treated sequence of the 250 bp upstream from the start codon of the *MeGI* gene was amplified by TaKaRa EpiTaq HS (TaKaRa) using sense- and antisense-specific primer set (Table 3).

### 4.6. Data Analysis

Data were expressed as means ± standard error (SE), and all RT-qPCR statistical analyses were analyzed by 2^−ΔΔCt^ method, where ΔΔCt = ((Ct, target (test) − Ct, target (control)) − ((Ct, reference (test) − Ct, reference (control)). Each experiment consisted of three technical replicates per condition.

## 5. Conclusions

Collectively, *OGI* is indispensable and low-expressed in androecious *D. kaki*, and the *Kali* located in the *OGI* promoter region exhibited high methylation level. On the other hand, it is certain that methylation levels of *MeGI* promoter may have important influence on the male flowers formation. Overall, our result was similar with the gender theory model of monoecious *D. kaki*. However, the molecular mechanism of unisexual flower formation in androecious resources of *D. kaki* remains largely elusive, and further assessment using high-sensitivity and -specificity detection methods will provide valuable knowledge for us to hopefully identify a potential candidate regulatory factor (may be siRNAs), apart from the *OGI*, to uncover the reason for male phenotype.

## Figures and Tables

**Table 1:** Details of the 87 samples investigated in this study.

No.	Sample Name ^a^	Sexuality ^b^	Genomic DNA PCR Amplification ^c^				Origin	Collection Site ^d^	RT-qPCR	Cloning and Sequencing	Bisulfite PCR Sequencing
			*Kali*	pOGI-Kali	*OGI*	*MeGI*					
1	Yunjiashan-1	A	+	+	+	+	Hubei Province	I	X		
2	Yunjiashan-3	A	+	+	+	+	Hubei Province	I	X	X	X (for Figure 2b)
3	Yunjiashan-5	A	+	+	+	+	Hubei Province	I	X		
4	Yunjiashan-6	A	+	+	+	+	Hubei Province	I	X		
5	Yunjiashan-7	A	+	+	+	+	Hubei Province	I	X		
6	Yunjiashan-8	A	+	+	+	+	Hubei Province	I	X	X	
7	Yunjiashan-14	A	+	+	+	+	Hubei Province	I	X		
8	Yunjiashan-16	A	+	+	+	+	Hubei Province	I	X		
9	Mulan-2	A	+	+	+	+	Hubei Province	II	X		
10	Mulan-3	A	+	+	+	+	Hubei Province	II	X		
11	Mulan-5	A	+	+	+	+	Hubei Province	II	X		
12	Mulan-7	A	+	+	+	+	Hubei Province	II	X		X (for Figure 3b)
13	Mulan-10	A	+	+	+	+	Hubei Province	II	X		
14	Mulan-14	A	+	+	+	+	Hubei Province	II	X		
15	Mulan-15	A	+	+	+	+	Hubei Province	II	X		
16	Mulan-16	A	+	+	+	+	Hubei Province	II	X		
17	Mulan-17	A	+	+	+	+	Hubei Province	II	X		
18	Mulan-21	A	+	+	+	+	Hubei Province	II	X		
19	Mulan-24	A	+	+	+	+	Hubei Province	II	X		X (for Figure 3b)
20	Mulan-30	A	+	+	+	+	Hubei Province	II	X		
21	Mulan-34	A	+	+	+	+	Hubei Province	II	X		
22	Mulan-38	A	+	+	+	+	Hubei Province	II	X	X	
23	Mulan-39	A	NT	NT	NT	NT	Hubei Province	II	X		
24	Mulan-40	A	+	+	+	+	Hubei Province	II			
25	Mulan-43	A	+	+	+	+	Hubei Province	II	X		
26	Mulan-44	A	+	+	+	+	Hubei Province	II	X		
27	Luotian Yeshi-1	A	+	+	+	+	Hubei Province	II	X	X	
28	Luotian Yeshi-2	A	+	+	+	+	Hubei Province	II			
29	Luotian Yeshi-17	A	+	+	+	+	Hubei Province	II			
30	Jiangsu Yeshi 1	A	+	+	+	+	Jiangsu Province	III	X		
31	Jiangsu Yeshi 1-2	A	+	+	+	+	Jiangsu Province	III	X		
32	Jiangsu Yeshi 1-3	A	+	+	+	+	Jiangsu Province	III			
33	Jiangsu Yeshi 1-7	A	+	+	+	+	Jiangsu Province	III	X		
34	Jiangsu Yeshi 1-10	A	+	+	+	+	Jiangsu Province	III	X		
35	Jiangsu Yeshi 2	A	+	+	+	+	Jiangsu Province	III	X	X	
36	Jiangsu Yeshi 2-1	A	+	+	+	+	Jiangsu Province	III	X		
37	Jiangsu Yeshi 2-2	A	+	+	+	+	Jiangsu Province	III			
38	Jiangsu Yeshi 2-4	A	+	+	+	+	Jiangsu Province	III	X		
39	Jiangsu Yeshi 2-20	A	+	+	+	+	Jiangsu Province	III			
40	Jiangsu Yeshi 2-48	A	NT	NT	NT	NT	Jiangsu Province	III	X		
41	Hunan Yeshi-1	A	+	+	+	+	Hunan Province	III	X	X	
42	Hunan Yeshi-2	A	+	+	+	+	Hunan Province	III	X		
43	Hunan Yeshi-3	A	+	+	+	+	Hunan Province	III	X		
44	Pingshanse-male	A	+	+	+	+	Hebei Province	III	X		
45	Jiangxi Yeshi-1	A	+	+	+	+	Jiangxi Province	III		X	X (for Figure 2c)
46	Jiangxi Yeshi-2	A	+	+	+	+	Jiangxi Province	III	X		
47	Jiangxi Yeshi-3	A	+	+	+	+	Jiangxi Province	III	X		
48	Jiangxi Yeshi-4	A	+	+	+	+	Jiangxi Province	III	X		
49	Jiangxi Yeshi-5	A	NT	NT	NT	NT	Jiangxi Province	III	X		
50	Qinghua-male	A	+	+	+	+	Shaanxi Province	IV	X		
51	Zhanfanghou-male	A	+	+	+	+	Shaanxi Province	IV	X		
52	Guangxi Yeshi-24	A	NT	NT	NT	NT	Guangxi Zhuang Autonomous Region	V	X		
53	Guangxi Yeshi-27	A	+	+	+	+	Guangxi Zhuang Autonomous Region	V	X		
54	Guangxi Yeshi-28	A	+	+	+	+	Guangxi Zhuang Autonomous Region	V	X		
55	Guangxi Yeshi-41	A	NT	NT	NT	NT	Guangxi Zhuang Autonomous Region	V	X		
56	Guangxi Yeshi-42	A	NT	NT	NT	NT	Guangxi Zhuang Autonomous Region	V	X		
57	Yaoxian Wuhuashi	M	+	+	+	+	Shaanxi Province	IV	X		
58	Heixinshi	M	+	+	+	+	Shaanxi Province	IV	X		
59	Xingyi Shuishi	M	+	+	+	+	Guizhou Province	IV	X		
60	Panxian Shuishi	M	+	+	+	+	Guizhou province	IV			
61	Shutouhong	M	+	+	+	+	Jiangsu Province	IV	X		
62	Xiangyang Niuxinshi	M	+	+	+	+	Hubei Province	IV	X		
63	Taiwan Zhengshi	M	+	+	+	+	Taiwan	IV	X		
64	Xiaobahu	M	NT	NT	NT	NT	Henan Province	III	X		
65	Laojianshan-5	M	+	+	+	+	Yunnan Province	VI	X		
66	Shougatsu	M	+	+	+	+	Japan	IV	X		
67	Zenjimaru	M	NT	NT	NT	NT	Japan	IV	X		
68	Okugosho	M	NT	NT	NT	NT	Japan	IV	X		
69	Hanagosho	M	NT	NT	NT	NT	Japan	IV	X		
70	Taishu	M	+	+	+	+	Japan	IV	X		
71	Nishimurawase	M	NT	NT	NT	NT	Japan	IV	X		
72	Jiangxi Yeshi-11	G	+	-	-	NT	Jiangxi Province	III			
73	Jiangsu Yeshi 1-1	G	+	-	-	NT	Jiangsu Province	III			
74	Xinan Niuxinshi	G	+	-	-	NT	Henan Province	III			
75	Luotian Yeshi-38	G	+	-	-	NT	Hubei Province	III			
76	Tianbaogai	G	+	-	-	NT	Hubei Province	VII			
77	Guangxi Yeshi-24	G	+	-	-	NT	Guangxi Zhuang Autonomous Region	V			
78	Luoanchuan Niuxin	G	+	-	-	NT	Henan Province	III			
79	Yangshuo Niuxin	G	+	-	-	NT	Yunan Province	III			
80	Huaxian Qingxuan	G	+	-	-	NT	Shaanxi Province	IV			
81	Boai Bayuehuang	G	+	-	-	NT	Henan Province	III			
82	Mopanshi	G	+	-	-	NT	Henan Province	III			
83	Sanyuan Jixinhusng	G	+	-	-	NT	Shaanxi Province	IV			
84	Huojing	G	+	-	-	NT	Shaanxi Province	IV			
85	Haian Xiaofangshi	G	+	-	-	NT	Shaanxi Province	IV			
86	Fuping Jianshi	G	+	-	-	NT	Shaanxi Province	IV			
87	Niutoushi	G	+	-	-	NT	Zhejiang Province	III			

^a^ All samples are *Diospyros kaki* Thunb. ^b^ Sexuality are represented by A: androecious sample; M: monoecious sample; G: gynoecious sample. ^c^ The electrophoresis bands of PCR product were indicated by + (positive) or - (negative); NT: not tested. ^d^ Samples were collected from I: Yunjia Mountain; II: Mulan Mountain; III: Yuanyang County, Henan Province; IV: Yangling District, Shaanxi Province, Yuanyang County, Henan Province; V: Guilin City, Guangxi Zhuang Autonomous Region; VI: Yuxi City, Yunnan Province; VII: Luotian County, Hubei Province.

**Table 2:** Types of termination codons and compositions of bases in *OGI* sequences derived from androecious *D. kaki*.

Name	Length (bp)	Termination Codon				Base Composition			
		TAG	TAA	TGA	Total (%)	A (%)	T (%)	G (%)	C (%)
*OGI* 1	918	21	21	12	54 (17.65)	298 (32.46)	234 (25.49)	205 (22.33)	181 (19.72)
*OGI* 2	947	23	20	13	56 (17.74)	305 (32.21)	238 (25.13)	213 (22.49)	191 (20.17)
*OGI* 3	913	22	21	13	56 (18.40)	296 (32.42)	230 (25.19)	203 (22.23)	184 (20.15)
*OGI* 4	913	22	21	13	56 (18.40)	298 (32.64)	232 (25.41)	203 (22.23)	180 (19.72)
*OGI* 5	909	22	21	13	56 (18.48)	298 (32.78)	232 (25.52)	203 (22.33)	176 (19.36)
*OGI* 6	928	21	20	14	55 (18.78)	302 (32.54)	242 (26.08)	204 (21.98)	180 (19.40)
*OGI* 7	910	23	21	13	57 (18.79)	293 (32.20)	231 (25.38)	204 (22.42)	181 (20.00)

*OGI* 1–7 represent the *OGI* sequence of Yunjiashan-8, Mulan-38, Luotian Yeshi-1, Jiangsu Yeshi 2, Jiangxi Yeshi-1, Hunan Yeshi-1, and Yunjiashan-3, respectively.

**Table 3:** Sequences of primers used in this study.

Purpose	Primer	Forward Primer Sequence (5′–3′)	Reverse Primer Sequence (5′–3′)
RT-qPCR	*GAPDH*	AGCTCTTCCACCTCTCCAGT	TGCTAGCTGCACAACCAACT
	*MeGI*	GGAGTTGAACTTTGGGAACG	AAGGCGACACTTGTGGACGA
	*OGI* ^a^	AACCCCATCGCATTTGATAA	CCGTCAATTTTGAGGGAGAG
PCR	*Kal i* ^b^	AACTGCCCAGGGGTACAACTAAG	TATTATTATGCTCCAACACTCGCAC
	*OGI* ^b^	CACAGTAGTCATATATTTTTAGC	CTGGCACACAAAATATTTTCAACCCT
	pOGI-*Kali* ^b^	CACCAAGTATTGATTTTTATTGTACCATTGCTTAT	TATTATTATGCTCCAACACTCGCAC
	*MeGI*	GGAGTTGAACTTTGGGAACG	AAGGCGACACTTGTGGACGA
Clone sequencing	*Kali* ^b^	AACTGCCCAGGGGTACAACTAAG	TATTATTATGCTCCAACACTCGCAC
	*OGI* ^b^	CACAGTAGTCATATATTTTTAGC	CTGGCACACAAAATATTTTCAACCCT
	pOGI-*Kali* ^b^	CACCAAGTATTGATTTTTATTGTACCATTGCTTAT	TATTATTATGCTCCAACACTCGCAC
	OGI-prom-gene	AGTGGATCCATCAAGGGTGC	GTGTCGTCCAGTTCCGCTTA
Bisulfite PCR sequencing	*Kali*	AATTGTTTAGGGGTGTAATTAAGTG	TTTTTTTTATTATTATACTCCAACACTC
	OGI-prom-F1	GAGAAATTTAATGTAATTTGTGAGG	CATAACAAATCTCTTCCATAACTAAAC
	OGI-prom-F2	GTTATGGAAGAGATTTGTTATGTTG	TCCACTAACACTTACTAAAAACCAC
	OGI-prom-F3	GTGGTTTTTAGTAAGTGTTAGTGGA	ATTAAATAAACAACTATCTAACTTATCTTTAC
	MeGI-SenseProm-bis	GTGTTTTGGTTAAATTAAGTTAATTTAATG	CTTTAATCAAAAAATTAAAATTAACTATCATTTT
	MeGI-ASProm-bis	TGATGATTTTTAATTGGAGGATTAAAGTTGGTTG	TCCCTCCATCCTCCCCAACAACACC

^a^ primer was provided by Akagi et al. [8]. ^b^ primers were provided by Akagi et al. [9].

## Data Availability

The data presented in this study are available on request from the corresponding author.

## Supplementary Materials

The following are available online at https://www.mdpi.com/2223-7747/10/2/390/s1, Figure S1: Alignment of the pOGI-Kali sequences. File S1: DNA sequences amplified by the primer of OGI-prom-gene.
