# Supplementary material for: Study of Sexual-Linked Genes (OGI and MeGI) on the Performance of Androecious Persimmons (Diospyros kaki Thunb.)
Source: Plants (Basel). 2021 Feb 18;10(2):390. doi: 10.3390/plants10020390 (PMC7922513; doi:10.3390/plants10020390)
Supplement: Supplementary file 1 [file plants-10-00390-s001.pdf]

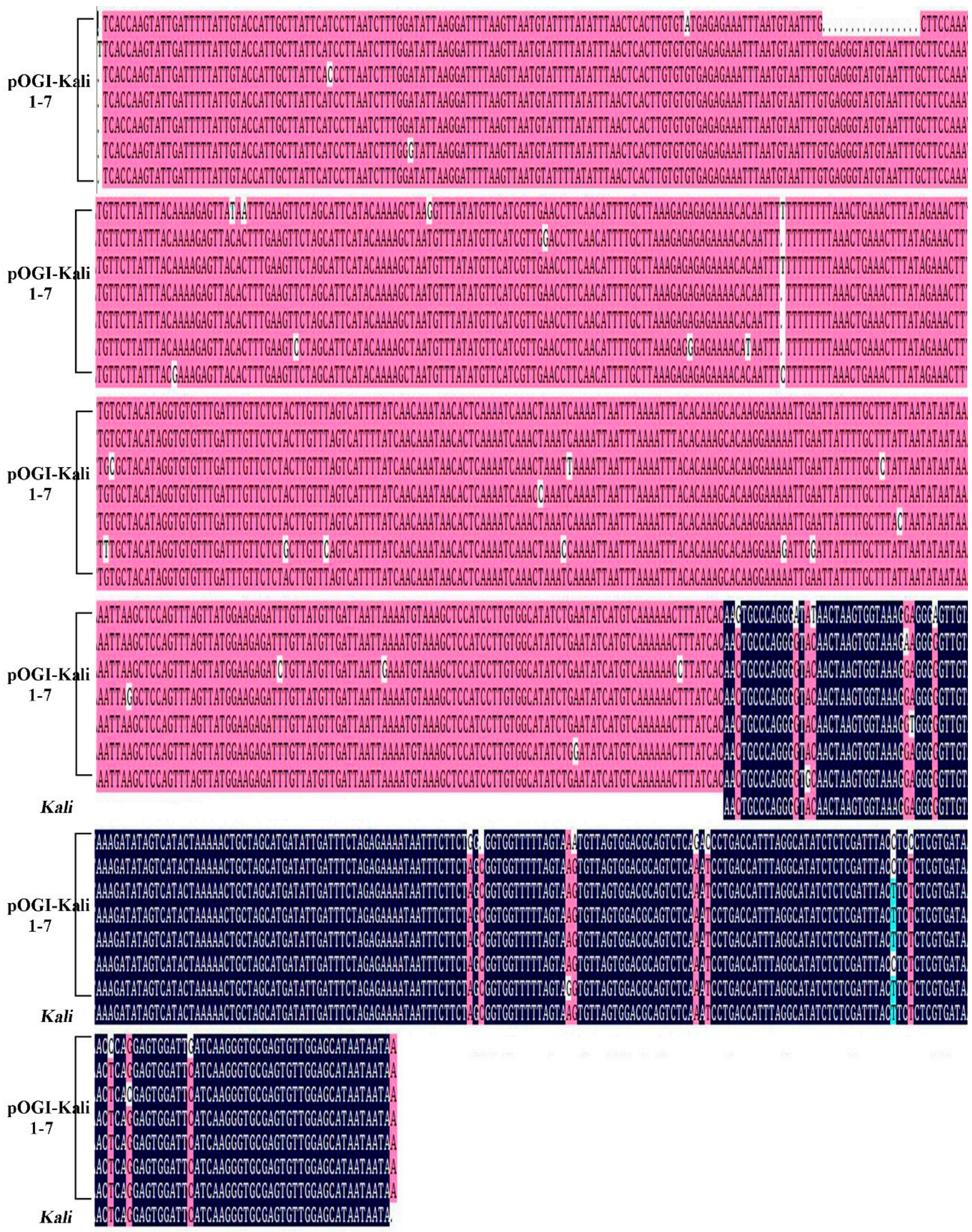

Figure S1. Alignment of the pOGI-Kali sequences. pOGI-Kali 1-7 represent the pOGI-Kali sequence of Yunjiashan-8, Mulan-38, Luotian Yeshi-1, Jiangsu Yeshi 2, Jiangxi Yeshi-1, Hunan Yeshi-1, and Yunjiashan-3, respectively, which were all obtained from our androecious *D. kaki*, and the 'Kali' sequence was from monoecious genotype, i.e., Taishu, from Akagi et al. [8].

File S1. DNA sequences amplified by the primer of OGI-prom-gene.

>Yunjiashan-8

TAGTGGATCCATCAAGGGTGCGGGTGTTGGAGCATAATAATAAAAAAAAAAAATTTATCAC
ATAACTTTTGGGAATCAACAACCAACGATAAGAAGCATTGAGATTTGTGCAAAAAAAAAC
AAGTGATGTGAACGTTGGCAATTCCGACTTCTGGTGAAATGTCAAATTCCACTGAAACACT
TGAATAGAGATAACGCAAAGATAAGCTAGATAGCTGTCCATTTAACTAGATCTTAGTCACC
CTCTAAAAAAAATAAATGCCAGAGATATTTAGTATTACACACATAATGCAAGCGGATACA
CACACAGATATATATATATATATATATATACCATATAAGGTTAACCCATTCCATGCCAGTAATG
TGACTATTTTCTGGCACACAAAATATTTTCAACCCTTGCACCCCAAACTCGCAGAGAAAAG
GTGGGTGCATGGCTTCCATTGAAGGGGTAGACCCCGTTAGGCTGTTGATCCAAACCCCATC
GCATTTGATAAGCTCCAATAGCCTCTGCTTCTCTTTCTCTGCCTCACTCAGCTGTTCCTTGAA
CTTTAGCACTTATCCAACACAGTAGTCATATAATTTTAGCTAACTGGTTTATGTGTGTCCCTT
AGAAACTCAATAGAGGCGGCACGAACATTTGTGTCTCTCCCTCAAAATTGACGGCCAATTT
TAATCCTCAGATTAAAGATCATCAAATGGATCTCATCTCTCATCAGTTCTTTCTAGTTGGGA
CATACAACTAAGCGGAACTGGACGACACA

>Mulan-38

TAGTGGATCCATCAAGGGTGCGAGTGTTGGAGCATAATAATAAAAAAAAAAAAATTTATCA
CATAACTTTTGGGAATCAACAAACAACGATAAGAAGCATTGAGATTTGTGCAAAAAAAAAC
AAGTGATGTGAACGTTGGCAATTCCGACTTCTGGTGAAATGTCAAATTCCACTGAAACACT
TGAATAGAGATAACGCAAAGATAAGCTAGATAGCTGTCCATTTAACTAGATCTTAGTCACC
CTCTAAAAAAAATAAATGCCAGAGATATTTAGTATTACACACACAATGCAAGCGGATACA
CACACAGATATATATATATATATATATATATATATATATACCATATAAGGTTAACCCATTCCAT
GCCAGTAATGTGACTATTTTCTGGCACACAAAATATTTTCAACCCTTGCACCCCAAACTCGC
AGAGAAAAGGTGGGTGCGTGGCTTCCATTGAAGGGGTAGACCTCATTAGGCTGTTGATCCA
AACCCCATCGCATTTGATAAGCTCCAATAGCCTCTGCTTCTCTTTCTCTGCCTCACTCAGCTG
TTCCTTGAACTTTAGCACTTATCCAACACAGTAGTCATATAATTTTAGCTAACTGGTTTATGT
GTGTCCCTTAGAAACTCAATAGAGGCGGCACGAACATTTGTGTCTCTCCCTCAAAATTGAC
GGCCAATTTTAATCCTCAGATTAAAGATCATCAAATGGATCTCATCTCTCATCAGTTCTTTCT
AGTTGGGACATACAACTAAGCGGAACTGGACGACACA

> Luotian Yeshi-1

TAGTGGATCCATCAAGGGTGCGAGTGTTGGAGCATAATAATAAAAAAAAAAAAATTTATCAC
ATAACTTTTGGGAATCAACAACCAACGATAAGAAGCATTGAGATTTGTGCAAAAAAAAACAA
GTGATGTGAACGTTGGCAATTCCGACTTCTGGTGAAATGTCAAATTCCACTGAAACACTTGA
ATAGAGATAACGCAAAGATAAGCTAGATAGCTGTCCATTTAACTAGATCTTAGTCACCCTCTA
AAAAAAATAAATGCCAGAGATATTTAGTATTACACACATAATGCAAGCGGATACACACACA

GATATATATATATATATATATATACCATATAAGGTTAACCCATTCCATGCCAGTAATGTGACTATTT
TCTGGCACACAAAATATTTTCAACCCTTGCACCCCAAACTCGCAGAGAAAAGGTGGGTGCG
CGGCTTCCATTGAAGGGGTAGACCTCATTAGGCTGTTGATCCAAACCCCATCGCATTTGATA
AGCTCCAATAGCCTCTGCTTCTCTTTCTCTGCCTCACTCAGCTGTTCCTTGAACTTTAGCACTT

ATCCAACACAGTAGTCATATAATTTTAGCTAACTGGTTTATGTGTGTCCCTTAGAAACTCAAT
AGAGGCGGCACGAACATTTGTGTCTCTCCCTCAAAATTGACGGCCAATTTTAATCCTCAGAT
TAAAGATCATCAAATGGATCTCATCTCTCATCAGTTCTTTCTAGTTGGGACATACAACTAAGC
GGAACTGGACGACACA

>Jiangsu Yeshi 2

TAGTGGATCCATCAAGGGTGCGAGTGTTGGAGCATAATAATAAAAAAAAAAAATTTATCACAT
AACTTTTGGGAATCAACAACCAACGATAAGAAGCATTGAGATTTGTGCAAAAAAAACAAGT
GATGTGAACGTTGGCAATTCCGACTTCTGGTGAAATGTCAAATTCCACTGAAACACTTGAAT
AGAGATAACGCAAAGATAAGCTAGATAGCTGTCCATTTAACTAGATCTTAGTCACCCTCTAA
AAAAAATAAATGCCAGAGATATTTAGTATTACACACATAATGCAAGCGGATACACACACAG
ATATATATATATATATATATATATATACCATATAAGGTTAACCCATTCCATGCCAGTAATGTGACT
ATTTTCTGGCACACAAAATATTTTCAACCCTTGCACCCCAAACTCGCAGAGAAAAGGTGGGT
GCGCGGCTTCCATTGAAGGGGTAGACCTCATTAGGCTGTTGATCCAAACCCCATCGCATTTG
ATAAGCTCCAATAGCCTCTGCTTCTCTTTCTCTGCCTCACTCAGCTGTTCCTTGAACTTTAGCA
CTTATCCAACACAGTAGTCATATAATTTTAGCTAACTGGTTTATGTGTGTCCCTTAGAAACTCA
ATAGAGGCGGCACGAACATTTGTGTCTCTCCCTCAAAATTGACGGCCAATTTTAATCCTCAG
ATTAAAGATCATCAAATGGATCTCATCTCTCATCAGTTCTTTCTAGTTGGGACATACAACTAA
GCGGAACTGGACGACACA

>Jiangxi Yeshi-1

TAGTGGATCCATCAAGGGTGCGAGTGTTGGAGCATAATAATAAAAAAAAAAAAATTTATCAC
ATAACTTTTGGGAATCAACAACCAACGATAAGAAGCATTGAGATTTGTGCAAAAAAATCAA
GTGATGTGAACGTTGGCAATTCCGACTTCTGTGAAATGTCAAATTCCACTGAAACACTTGAA
TAGAGATAACGCAAAGATAAGCTAGATAGCTGTCCATTTAACTAGATCTTAGTCACCCTCTAA
AAAAAATAAATGCCAGAGATATTTAGTATTACACACATAATGCAAGCGGATACACACACAG
ATATATATATATATATATATATATATATATACCATATAAGGTTAACCCATTCCATGCCAGTAATGTGA
CTATTTTCTGGCACACAAAATATTTTCAACCCTTGCACCCCAAACTCGCAGAGAAAAGGTGG
GTGCGCGGCTTCCATTGAAGGGGTAGACCTCATTAGGCTGTTGATCCAAACCCCATCGCATT
TGATAAGCTCCAATAGCCTCTGCTTCTCTTTCTCTGCCTCACTCAGCTGTTCCTTGAACTTT

AGCACTTATCCAACACAGTAGTCATATAATTTTAGCTAACTGGTTTATGTGTGTCCCTTAGAA
ACTCAATAGAGGCGGCACGAACATTTGTGTCTCTCCCTCAAAATTGACGGCCAATTTTAATC
CTCAGATTAAAGATCATCAAATGGATCTCATTTCTCATCAGTTCTTTCTAGTTGGGACATACA
ACTAAGCGGAACTGGACGACACA

>Hunan Yeshi-1

TAGTGGATCCATCAAGGGTGCGAGTGTTGAAGCATAATAATAAAAAAAAAAAAATTTATCAC
ATAACTTTTGGGAATCAACAACCAACGATAAGAAGCATTGAGATTTGTGCAAAAAAAAACAA
GTGATGTGAACGTTGGCAATTCCGACTTCTGGTGAAATGTCGAATTCCACTGAAACACTTGA
ATAGAGATAACGCAAAGATAAGCTAGATAGCTGTCCATTTAACTAGATCTTAGTCACCCTCTA
AAAAAAATAAATGCCAGAGATATTTAGTGTTACACACATAATGCAAGCGGATACACACACA
GATATATATATATATACCATATAAGGTTAACCCATTCCATGCCAGTAATGTGACTATTTTCTGGC
ACACAAAATATTTTCAACCCTTGCACCCCAAACTCGCAGAGAAAAGGTGGGTGCGTGGCTT
CCATTGAAGGGGTAGACCTCATTAGGCTGTTGATCCAAACCCCATCGCATTTGATAAGCTCC
AATAGCCTCTGCTTCTCTTTCTCTGCCTCACTCAGCTGTTCCTTGAACTTTAGCACTTATCCAA
CACAGTAGTCATATAATTTTAGCTAACTGGTTTATGTGTGTCCCTTAGAAACTCAATAGAGGC
GGCACGAACATTTGTGTCTCTCCCTCAAAATTGACGGCCAATTTTAATCCTCAGATTAAAGAT
CATCAAATGGATCTCATCTCTCATCAGTTCTTTCTAGTTGGGACATACAACTAAGCGGAACTG
GACGACACA

>Yunjiashan-3

TAGTGGATCCATCAAGGGTGCGAGTGTTGGAGCATAATAATAAAAAAAAAAAAATTTATCAC
ATAACTTTTGGGAATCAACAACCAACGATAAGAAGCATTGAGATTTGTGCAAAAAAAAACAA
GTGATGTGAACGTTGGCAATTCCGACTTCTGGTGAAATGTCAAATTCCACTGAAACACTTGA
ATAGAGATAACGCAAAGATAAGCTAGATAGCTGTCCATTTAACTAGATCTTAGTCACCCTCTA
AAAAAAATAAATGCCAGAGATATTTAGTATTACACACATAATGCAAGCGGATACACACACA
GATATATATATATATATATATATATATACCATATAAGGTTAACCCATTCCATGCCAGTAATGTGA
CTATTTTCTGGCACACAAAATATTTTCAACCCTTGCACCCCAAACTCGCAGAGAAAAGGTGG
GTGCGCGGCTTCCATTGAAGGGGTAGACCTCATTAGGCTGTTGATCCAAACCCCATCGCATT
TGATAAGCTCCAATAGCCTCTGCTTCTCTTTCTCTGCCTCACTCAGCTGTTCCTTGAACTTTAG
CACTTATCCAACACAGTAGTCATATAATTTTAGCTAACTGGTTTATGTGTGTCCCTTAGAAACT
CAATAGAGGCGGCACGAACATTTGTGTCTCTCCCTCAAAATTGACGGCCAATTTTAATCCTC
AGATTAAAGATCATCAAATGGATCTCATCTCTCATCAGTTCTTTCTAGTTGGGACATGCAACT
AAGCGGAACTGGACGACACA
